# Update in genetic and epigenetic causes of hypertension

**DOI:** 10.1007/s00018-024-05220-4

**Published:** 2024-04-30

**Authors:** Arya Mani

**Affiliations:** 1 Department of Internal Medicine, Yale University School of Medicine, Yale Cardiovascular Research Center, 300 George Street, New Haven, CT 06511 USA; 2 Department of Genetics, Yale School of Medicine, New Haven, CT USA

**Keywords:** Hypertension, Genetics, Epigenetics, Monogenic, Polygenic, GWAS, PRDM6, CELA2A, LRP6

## Abstract

Hypertension is a heritable disease that affects one-fourth of the population and accounts for about 50% of cardiovascular deaths. The genetic basis of hypertension is multifaceted, involving both monogenic and most commonly complex polygenic forms*.* With the advent of the human genome project, genome-wide association studies (GWAS) have identified a plethora of loci linked to hypertension by examining common genetic variations. It's notable, however, that the majority of these genetic variants do not affect the protein-coding sequences, posing a considerable obstacle in pinpointing the actual genes responsible for hypertension. Despite these challenges, precise mapping of GWAS-identified loci is emerging as a promising strategy to reveal novel genes and potential targets for the pharmacological management of blood pressure. This review provides insight into the monogenic and polygenic causes of hypertension. Special attention is given to PRDM6, among the earliest functionally characterized GWAS-identified genes. Moreover, this review delves into the roles of genes contributing to renal and vascular forms of hypertension, offering insights into their genetic and epigenetic mechanisms of action.

## Introduction

### Hypertension, a renal or vascular, genetic, or epigenetic disease: attempt to resolve the old debates

Hypertension is a global health issue, impacting more than one billion adults worldwide, with approximately one in every four adults of all ages in the United States affected by the condition. Among individuals aged 70 and older, around 75% are afflicted by hypertension, making it the primary cause of death in this age group. Notably, hypertension is the largest modifiable risk factor for cardiovascular diseases on a global scale, contributing to an estimated 50% of cardiovascular-related deaths [1]. Despite significant investments in the development of antihypertensive drugs, approximately 50% of individuals with hypertension are unable to achieve the target blood pressure levels [2], indicating an urgent need for the discovery of novel therapeutic targets.

Twin [3, 4], as well as genome-wide association studies (GWAS) [5], have indicated that blood pressure variation has a heritability of approximately 50%. While the initial genes identified for hypertension primarily underlie the monogenic form of the disease, it is important to recognize the broader contribution of genetic and environmental factors in its development.

Rare forms of autosomal dominant and at least one form of autosomal recessive inheritance of hypertension were the first reported cases that indicated its heritability. The discovery of the genetic underpinning of the monogenic form of hypertension has been very illuminating in understanding the disease pathogenesis and the identification of novel targets for their treatment. However, the identified genetic variants accounted for only a small fraction of heritability [6]. Early investigations on the heritability of hypertension indicated that blood pressure is a continuous trait with a complex mode of inheritance, influenced by both genetic predisposition and environmental factors [7]. With the advent of the human genome project and the explosion of genome-wide association studies this hypothesis has been substantiated, and to date, over 800 genetic loci that influence blood pressure have been identified. Each of these loci contributes to less than 1 mmHg of blood pressure variation [8]. These findings contradict the initial notion that hypertension is primarily a bimodal trait and therefore a disorder caused by a single gene [9].

Several decades before the first genetic discoveries, it was proposed that arterial pressure elevation stems from salt retention and expansion of extracellular fluid volume [10]. The genetic findings have confirmed the role of salt and water balance as the underlying mechanism for hypertension. Interestingly, the 'secondary' causes of hypertension such as primary aldosteronism, and Cushing's syndrome have often genetic causes and are associated with increased salt and water absorption in the distal nephron. However, most recent genetic findings have shown that hypertension can also be caused by abnormal function of endothelial [11] or vascular smooth muscle cells (VSMC) [12]. This review aims to present a comprehensive overview of the monogenic and polygenic causes of hypertension, highlighting their respective roles in renal and vascular hypertension, and exploring the mechanisms of action that range from genetic to epigenetic modulation of blood pressure traits.

Additionally, we will delve into the epigenetic regulation of blood pressure traits, focusing on the modulation of gene expression. A particular emphasis will be placed on PRDM6, an SMC-specific histone modifier, and its involvement in blood pressure regulation. We will examine its effects on the differentiation of renin-producing cells and its influence on other blood pressure genes identified through GWAS.

Furthermore, we will discuss the genetics of hypertension in the context of metabolic syndrome, aiming to shed light on the interplay between the metabolic risk factors. By resolving longstanding debates surrounding the role of the kidney versus vasculature in hypertension, this review seeks to provide new insights into the pathogenesis and treatment of this complex disease.

### The monogenic forms of hypertension and their role in increased salt and water retention (Fig. 1)

**Fig. 1:**
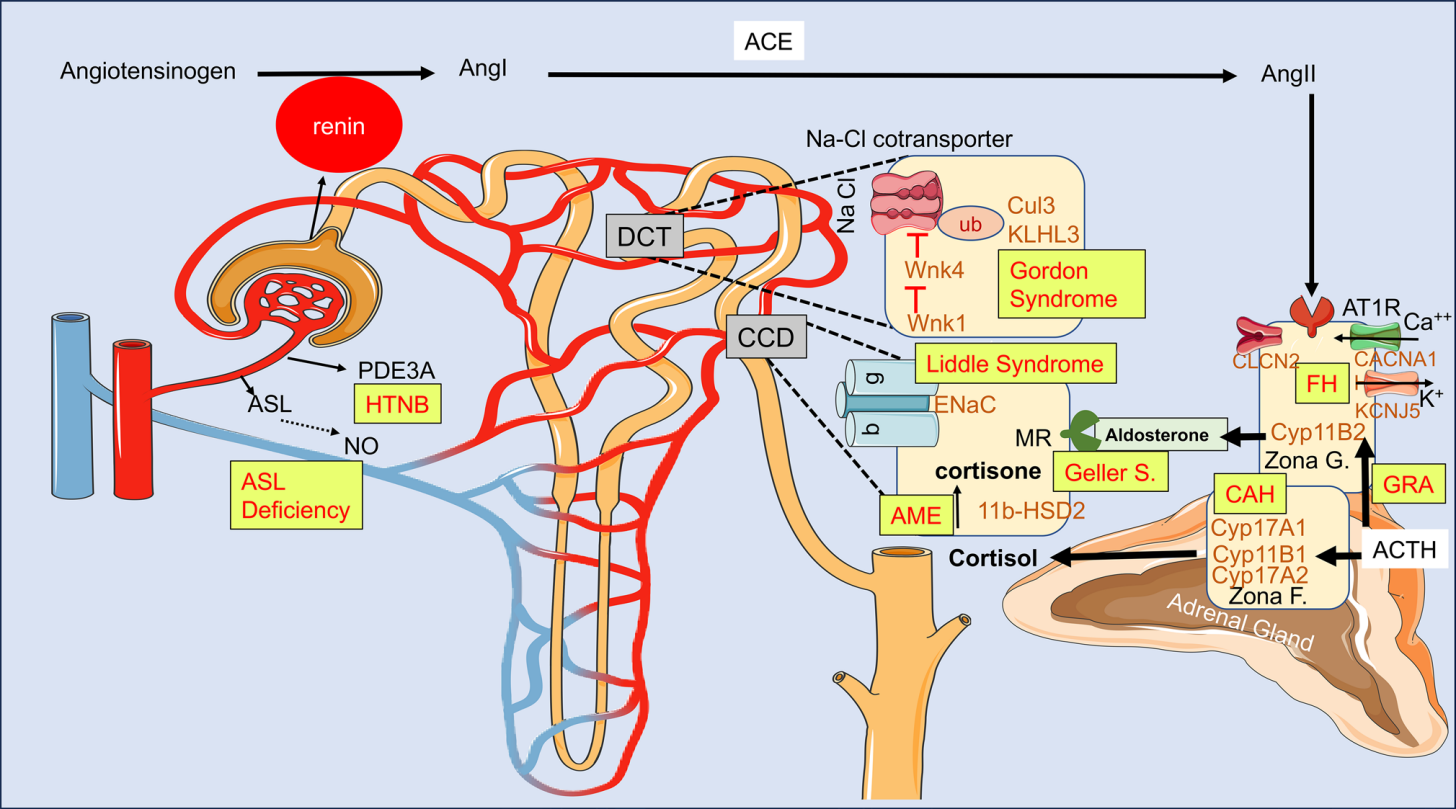
Monogenic causes of hypertension and their effect on renal handling of salt and water, vasculature endothelial, and smooth muscle cell differentiation

*Glucocorticoid-remediable aldosteronism (GRA)*, also known as familial hyperaldosteronism (FH) type I, is a syndrome characterized by autosomal dominant hypertension. It is caused by a chromosomal rearrangement that results in a hormonal defect in the adrenal gland. Specifically, this defect arises from the fusion of the promoter region of the *11β-hydroxylase* gene (*CYP11B1*) with the coding regions of the aldosterone synthase gene (*CYP11B2*) on chromosome 8q. Because of this genetic abnormality, the production of aldosterone becomes activated by adrenocorticotropic hormone (ACTH), independent of the renin–angiotensin–aldosterone system (RAAS) pathway activation. This dysregulation leads to hyperaldosteronism, salt, and water retention, leading to hypertension [13]. The renin levels are suppressed and are characteristically low in this condition.

Patients diagnosed with glucocorticoid-remediable aldosteronism (GRA) commonly exhibit early-onset hypertension and may experience mild hypokalemia, and metabolic alkalosis. The diagnosis is often established when significant hypokalemia occurs after treatment with thiazide diuretics. Furthermore, individuals with GRA face an elevated risk of intracranial bleeding [14], which is why brain imaging is recommended for screening at the onset of puberty. Confirming the diagnosis involves administering small doses of glucocorticoids, which inhibit the ACTH-aldosterone axis and effectively resolve hypertension [15].

In summary, GRA is a rare genetic disorder that should be considered in patients presenting with early-onset hypertension, hypokalemia, metabolic alkalosis, and low renin levels. The administration of low doses of glucocorticoids has proven to be an effective treatment strategy for controlling hypertension and preventing the onset of associated complications.

*Familial Hyperaldosteronism (FH)* FH is a genetic condition primarily attributed to autosomal dominant aldosterone-producing adenomas or hyperplasia. Unlike GRA, the hypertension associated with FH does not respond to glucocorticoids. FH encompasses various subtypes, with FH type II being characterized by heterozygous gain-of-function mutations in the *CLCN2* gene, encoding a voltage-gated chloride channel expressed in adrenal zona glomerulosa cells. Both inherited and de-novo mutations have been reported. The presence of mutant channels increases the minimum open probability or slows down the deactivation of the gates, resulting in significantly larger chloride efflux compared to wildtype. This in turn induces the expression of aldosterone synthase, encoded by the *CYP11B2* gene and its upstream regulator NR4A2 (Fig. 1). These mutant channels facilitate enhanced channel opening even at resting potentials [16]. FH type III is attributed to gain-of-function mutations in the *KCNJ5* gene, encoding the inward rectifying potassium channel, KCNJ5 [17]. These mutations can disrupt membrane ion selectivity, leading to membrane depolarization and increased calcium entry into the adrenal glomerulosa cells. As a result, hypertension in early childhood, adrenal hyperplasia, hyperaldosteronism, and severe hypokalemia may occur. Patients also have elevated levels of the hybrid steroids 18-oxocortisol and 18-hydroxycortisol. The hypertension and aldosteronism in FH type III are not reversed by administration of exogenous glucocorticoids and the treatment often necessitates bilateral adrenalectomy, particularly in cases where the condition proves to be unresponsive to drug therapy [18].

FH type IV is caused by inherited or de-novo gain-of-function missense mutations in the *CACNA1H* gene, which encodes a T-type calcium channel [19]. These mutations lead to altered channel activity, resulting in dysregulation of calcium influx. This dysregulation contributes to the pathogenesis of the condition, characterized by hyperaldosteronism in childhood.

*The congenital adrenal hyperplasia syndrome* Congenital adrenal hyperplasia (CAH) is caused by autosomal recessive mutations in the *CYP11B1* gene encoding steroid 11 β-hydroxylase, *CYP17A1* gene encoding 17 α-hydroxylase or *CYP17A2* gene encoding 21-hydroxylase (Fig. 1), resulting in androgen excess, virilization, and hypertension. Mutations in the *CYP17A2 gene account for 90% of cases.* Most mutations are large-scale deletions, conversions, or splice-site mutations. Mechanistically, the low plasma cortisol levels lead to a loss of feedback inhibition on the pituitary gland and result in increased ACTH production and adrenal hyperplasia. This results in the accumulation of cortisol precursors, causing hypokalemia, increased salt and water uptake, and subsequent hypertension via mineralocorticoid receptor activation [20]. Patients with *CYP11B1* mutations also present with precocious puberty and virilization due to excess androgenic sex hormone production. Treatment includes steroids to suppress the pituitary gland and mineralocorticoid receptor antagonists, such as spironolactone, for hypertension.

CAH caused by mutations in the *CYP17A1* gene, which also has 17,20-lyase activities is characterized by sex hormone synthesis blockade, resulting in delayed sexual development in girls and ambiguous genitalia (pseudohermaphroditism) in boys. The treatment involves the use of sex hormones in addition to steroids, and mineralocorticoid receptor antagonists for hypertension.

*Apparent mineralocorticoid excess (AME)* Apparent mineralocorticoid excess (AME) is a recessive disorder caused by a congenital defect of the renal in 11-beta-hydroxysteroid dehydrogenase type II (HSD11B2) isozyme, which normally metabolizes cortisol to cortisone and prevents cortisol from binding to the mineralocorticoid receptor. In the absence of 11-β HSD2 (Fig. 1), excess cortisol accumulates and binds to the mineralocorticoid receptor, resulting in sodium retention, volume expansion, and hypertension. This condition is characterized by low plasma renin activity, hypokalemia, metabolic alkalosis, hypercalciuria, and nephrocalcinosis [21, 22]. The severity of the disease depends on the levels of the 11-β HSD2 enzyme, which can vary among patients. Some patients may only experience mild hypertension, while others may develop early-onset severe hypertension and end-organ damage.

Treatment for AME involves the use of mineralocorticoid receptor antagonists such as spironolactone and eplerenone, as well as epithelial Na channel blockers like amiloride. Hypercalciuria can be managed with thiazide diuretics, with potassium supplementation as needed [20]. Blood pressure responds well to these treatments.

*Autosomal dominant, early-onset hypertension with severe exacerbation in pregnancy (Geller syndrome)* A phenocopy of mineralocorticoid excess syndrome, the disorder is characterized by hypertension exacerbated during pregnancy. The disease is inherited in an autosomal dominant manner and leads to early-onset hypertension, which is worsened during pregnancy due to the activation of the mineralocorticoid receptors by progesterone. Clinical features of this syndrome include low serum renin and aldosterone levels and a nonsignificant trend toward low plasma potassium levels. This extremely rare syndrome is caused by an activating mutation in the mineralocorticoid receptor gene MR, also known as NR3C2(nuclear receptor subfamily 3, group c, member 2). The mutation in a multiplex kindred with 11 affected individuals resulted in S810L substitution in the receptor, losing its specificity for aldosterone and its activation also by progesterone [23].

*Liddle syndrome* is an autosomal dominant condition caused by gain-of-function mutations in the *SCNN1A* [24], *SCNN1B* [25], and *SCNN1G* [26] genes, which encode the a, β, and γ subunits of the epithelial sodium channel ENaC. The mutations in *SCNN1B* and *SCNN1G* genes occur in the intracellular PY motif that interacts with the WW domain in NEDD4 [27]. These mutations impair the ubiquitination of ENaC subunits by NEDD4 and their subsequent proteasomal degradation, resulting in their accumulation on the cell surfaces of the cortical collecting tubules. This leads to increased sodium reabsorption, which can cause hypokalemia, metabolic alkalosis, low renin and aldosterone levels, and early onset salt-sensitive hypertension in patients with Liddle syndrome [28]. The mutations in the *SCNN1A* gene are extremely rare and occur in its extracellular domain, triggering its activation*.* The *SCNN1D* gene is minimally expressed in the kidney and may cause hypertension via inflammation in the kidney [29, 30].

*Pseudohypoaldosteronism type II (Gordon syndrome, familial hyperkalemic HTN)* Gordon syndrome or pseudohypoaldosteronism type II, also known as familial hyperkalemic hypertension is an autosomal dominant or recessive disorder, characterized by hypertension, hyperkalemia, and hyperchloremic metabolic acidosis. This syndrome is caused by mutations in 4 genes that include the serine-threonine kinases “With No lysine [K]” kinases (*WNK1* and *WNK4*) *KeLcH-Like3* and *CULlin3*. These encoded proteins collectively regulate the membrane expressions of the thiazide-sensitive sodium–chloride cotransporter (NCC) in the distal convoluted tubule and ROMK in the principal cells of the collecting duct. WNK1 and 4 localize to the distal convoluted tubule (DCT) and cortical collecting duct and are involved in the regulation of the sodium–chloride transporter acting via phosphorylation of oxidative stress-responsive gene 1 (OSR1)/Ste20-related proline–alanine-rich kinase (SPAK). WNK1 is cytoplasmic, while WNK4 localizes to tight junctions. Mutations in WNK1 and WNK4 increase WNK kinase activity, leading to augmented membrane expression of membrane NCC, reduced expression of the renal outer medullary potassium channel (ROMK), and altered activities of several other key transporters and channels, including the epithelial sodium channel (ENaC). This results in increased absorption of sodium and chloride, impacting fluid balance and blood pressure and reduced secretion of potassium. Disease-causing mutations in WNK1 include large intronic deletions that increase *WNK1* expression [31]. The mutations in *WNK4* cluster in a highly conserved segment of the encoded protein [31]. Mechanistically, WNK4 has been shown to downregulate the thiazide-sensitive NaCl cotransporter (NCC) activity, an effect that is suppressed by WNK1 [32]. The regulation of potassium secretion is complex and is likely multifactorial. In general, potassium secretion occurs in the collecting duct and is greatly dependent on the delivery of sodium. The uptake of sodium creates lumen negativity, which enables potassium secretion via ROMK. Increased sodium reabsorption in the DCT reduces sodium transport to the collecting duct, diminishing potassium secretion. In addition, WNK mutations reduce ROMK function by diminishing their membrane expression.

Pseudohypoaldosteronism type II is also caused by mutations in the *cullin-3* (*CUL3*) and *kelch-like 3* (*KLHL3*) genes [33]. Immunoprecipitation studies have shown that KLHL3 is strongly bound to WNK isoforms and CUL3. Further investigation revealed that the CUL3-KLHL3 E3 ligase-RING complex regulates blood pressure by ubiquitinating WNK isoforms. Failure of the complex to ubiquitinate WNK isoforms can trigger hypertension. KLHL3 ubiquitinates WNK1 and WNK4 and its loss of function can cause both recessive or dominant LOF mutations. The recessive form has earlier disease onset and more severe phenotypes. CUL3 mutations are dominant, either inherited or de novo, and cause gain-of-function by targeting *KLHL3* for degradation and preventing *WNK* degradation.

### The monogenic vascular hypertension

The number of discovered genes underlying the vascular form of hypertension compared to the genes causing water and salt retention is considerably lower. However, the recent discoveries of common variants by GWAS suggest a greater contribution of genes regulating vascular cell development and function to the development of hypertension.

*Argininosuccinate lyase (ASL)* deficiency is a prototypical form of inherited autosomal recessive endothelial-cell dependent hypertension [34]. The endothelium plays a critical role in the regulation of peripheral vascular resistance by modulating nitric oxide (NO) production. ASL is the fourth enzyme of the urea cycle that reversibly catalyzes the breakdown of argininosuccinate (ASA) and is involved in the biosynthesis of arginine and the production of urea. The impaired function of this gene results in inadequate urea and arginine production, causing endothelial dysfunction, leading to reduced NO production, increased oxidative stress, and impaired angiogenesis.

*Autosomal hypertension with brachydactyly type e (HTNB)* is another example of vascular hypertension that is associated with brachydactyly and death from stroke before the age of 50 [35]. The syndrome also features neurovascular contact at the rostral-ventrolateral medulla and altered baroreflex blood pressure regulation. It is a salt-resistant and age-dependent hypertension caused by autosomal dominant mutations of the phosphodiesterase *PDE3A* gene [11]. PDE3A is a member of the PDE family of enzymes, which degrades both cyclic adenosine monophosphate (cAMP) and cyclic guanosine monophosphate (cGMP). It has three isoforms that contain the same catalytic region but differ at their N terminus. *PDE3A2* [36] is the major isoform in human vascular smooth muscle cells (VSMCs) [36], *PDE3A1* is the major isoform in the heart [37] and *PDE3A3* is the major isoform in the placenta [38]. The HTNB mutations cause amino acid substitutions in PDE3A1 and A2 enzymes N-terminally of the catalytic domain.

The study of a human *PDE3A* mutation that causes an amino acid substitution within a 5 amino-acid-long PDE3A region N-terminally of the enzyme's catalytic domain has revealed that the mutations result in PKA- or PKC-mediated hyperphosphorylation of critical regulatory serine 428 and 438 sites in phosphodiesterase 3A and a gain of its cAMP-hydrolytic activity [11]. In vitro studies suggest that PDE3A inhibitors and cyclic guanosine monophosphate suppress the hyperactivity of the mutant proteins.

### Polygenic (essential) hypertension: the role of epigenetic factors and the connections to renovascular disease

Genome-wide discovery analyses targeting blood pressure (BP) characteristics such as systolic BP (SBP), diastolic BP (DBP), and pulse pressure (PP) have unearthed over 800 loci associated with these traits [39]. Despite this, the genetic variations discovered account for merely about 5% of the traits' heritability. Notably, most of these genetic variants are situated in intronic or intergenic regions, leaving the causal genes at these loci predominantly unidentified. An analysis of GWAS data from the Atherosclerosis Risk in Communities (ARIC) study, which included 8901 individuals of European ancestry and 2860 of African Ancestry, revealed that the heritability of blood pressure, with limited exceptions, originates from loci not associated with genes implicated in the monogenic forms of hypertension. This study also found that loci linked to BP and hypertension (HTN) explain merely ~ 5% of the heritability [5]. Functional characterization of the candidate genes has been rarely carried out. Common variants in the promoter region of the UMOD gene, responsible for uromodulin production—the primary protein in normal urine—are associated with increased UMOD expression [40]. This increase is linked to a higher susceptibility to chronic kidney disease and hypertension. Overexpression of uromodulin in transgenic mice resulted in salt-sensitive hypertension and age-dependent renal injury, mediated by the activation of the renal sodium cotransporter NKCC2. Notably, NKCC2 pharmacological inhibition showed enhanced efficacy in lowering blood pressure in hypertensive individuals carrying UMOD promoter risk variants compared to other hypertensive subjects.

A subsequent GWAS focusing on populations of East Asian, South Asian, and European descents investigated the global and population-specific genetic factors underlying five blood pressure traits. The study identified 12 new loci, including PRDM6 and phosphodiesterase 3A (PDE3A), the latter of which was known for its role in vascular hypertension [39]. The researchers reported an association between sequence variations, environmental factor-induced epigenetic changes such as DNA methylation near identified genes, and blood pressure variability. Further investigations into DNA methylation have shown its significant correlation with the onset and severity of hypertension. For instance, lower levels of 5-methylcytosine (5mC) have been linked to advanced stages of hypertension in patients with essential HTN, and hypomethylation of the angiotensin II type I receptor gene has been associated with higher SBP and DBP levels. Hypertensive smokers, in particular, demonstrated reduced methylation levels.

A comprehensive GWAS involving one million Europeans explored the linkage between sentinel single nucleotide polymorphisms (SNPs) at 901 loci and lifestyle traits within the UK Biobank, unveiling 535 previously unidentified blood pressure loci. This study illuminated the shared genetic architecture between blood pressure and lifestyle factors, identifying significant associations between daily consumption of fruits, body mass, water, caffeine, tea intake, and BP-related genetic variants [41].

### Common PRDM6 variants: intersection of renal and vascular determinants of hypertension

A recent study by our group identified *PRDM6* (PRDI-BF1 and RIZ homology domain-containing protein 6) as the causal gene for hypertension at the 5q23 locus using massively parallel reporter and gene editing assays [12].

Prdm6 locus had emerged as a significant player in blood pressure traits based on findings from several genome-wide association studies (GWAS) in diverse populations. Prdm6 is initially expressed in vascular precursor cells [42]. The presence of PRDM6 alters the fate of these cells towards smooth muscle cells. As a result, its highest expression levels in adults are found in the smooth muscle cells within the aorta and other arteries, rendering it an appealing candidate for blood pressure regulation. Furthermore, it is a histone modifier [39, 43] and by this nature can affect the expression of numerous genes. As an epigenetic regulator, it also has the potential to mediate the effects of environment such as nutrition on blood pressure. Through the utilization of massively parallel reporter assays and CRISPR-mediated deletions of single nucleotide polymorphisms (SNPs) associated with blood pressure, our research team has successfully identified multiple causal SNPs derived from GWAS within the third intron of the *Prdm6* gene [12]. These SNPs, both individually and in a combinatorial fashion, demonstrated allele-specific expression effects on Prdm6. Notably, this investigation uncovered a super-enhancer region that harbors multiple transcription factor binding sites, such as STAT1, RBP-J, SMAD3, and serum response factor (SRF). The activation of STAT1 by IFNg increased PRDM6 expression in human embryonic kidney (HEK1) cells. Accordingly, the CRISPR-Cas mediated deletion of the enhancer region containing STAT1 binding regions led to a significant reduction in Prdm6 expression. This suggests that the interaction of these transcription factors with the enhancer region is crucial for the regulation of Prdm6 expression.

Homozygous deletion of Prdm6 in mice expressing Cre recombinase under the control of the mouse smooth muscle cell protein 22-α promoter (Prdm6fl/fl SM22-Cre) leads to death caused by patent ductus arteriosus shortly after birth [44]. Remarkably, when PRDM6 was inducibly deleted in mice expressing tamoxifen-inducible Cre under the control of the mouse smooth muscle *myosin heavy polypeptide 11* (*Myh11*) promoter/enhancer, no changes in blood pressure were observed. Strikingly, mice with heterozygous SMC deletion of PRDM6 (Prdm6fl/ + SM22-Cre) developed hypertension when subjected to a high salt diet. This finding strongly suggests that PRDM6 plays a developmental role in blood pressure regulation. The increase in blood pressure correlated with elevated expression levels of renin in the kidneys and aldosterone synthase in the adrenal glands. The mechanistic explanation involves the absence of PRDM6, which leads to heightened levels of *Sox6* transcripts. This, in turn, disrupts the differentiation process of renin-producing cells, causing them to lose their ability to develop into smooth muscle cells. Normally, renin-producing cells are distributed throughout the embryonic kidney along the renal vasculature. However, when these cells differentiate into smooth muscle cells, they become restricted to the juxtaglomerular apparatus, which is located near the macula densa of the distal renal tubule. In this specialized location, they can sense sodium levels in the urine and secrete renin when sodium is low. The loss of differentiation in renin-producing cells and the subsequent increase in their population results in elevated levels of plasma renin and the development of hypertension.

Consistent with these findings, mice with smooth muscle cell-specific Sox6 knockout exhibited very few renin-producing cells. Accordingly, the expression of renin significantly decreased in Prdm6fl/fl SM22-Cre mice after Sox6 deletion. Furthermore, the blood pressure of Prdm6fl/ + SM22-Cre mice returned to normal when treated with the renin inhibitor aliskiren. Interestingly, transcriptome analysis of the aorta revealed that PRDM6 regulates 46 genes located at GWAS peak loci associated with hypertension, suggesting that PRDM6 acts as a driver of a blood pressure gene regulatory network and represents an attractive target for the development of antihypertensive drugs.

**Fig. 2:**
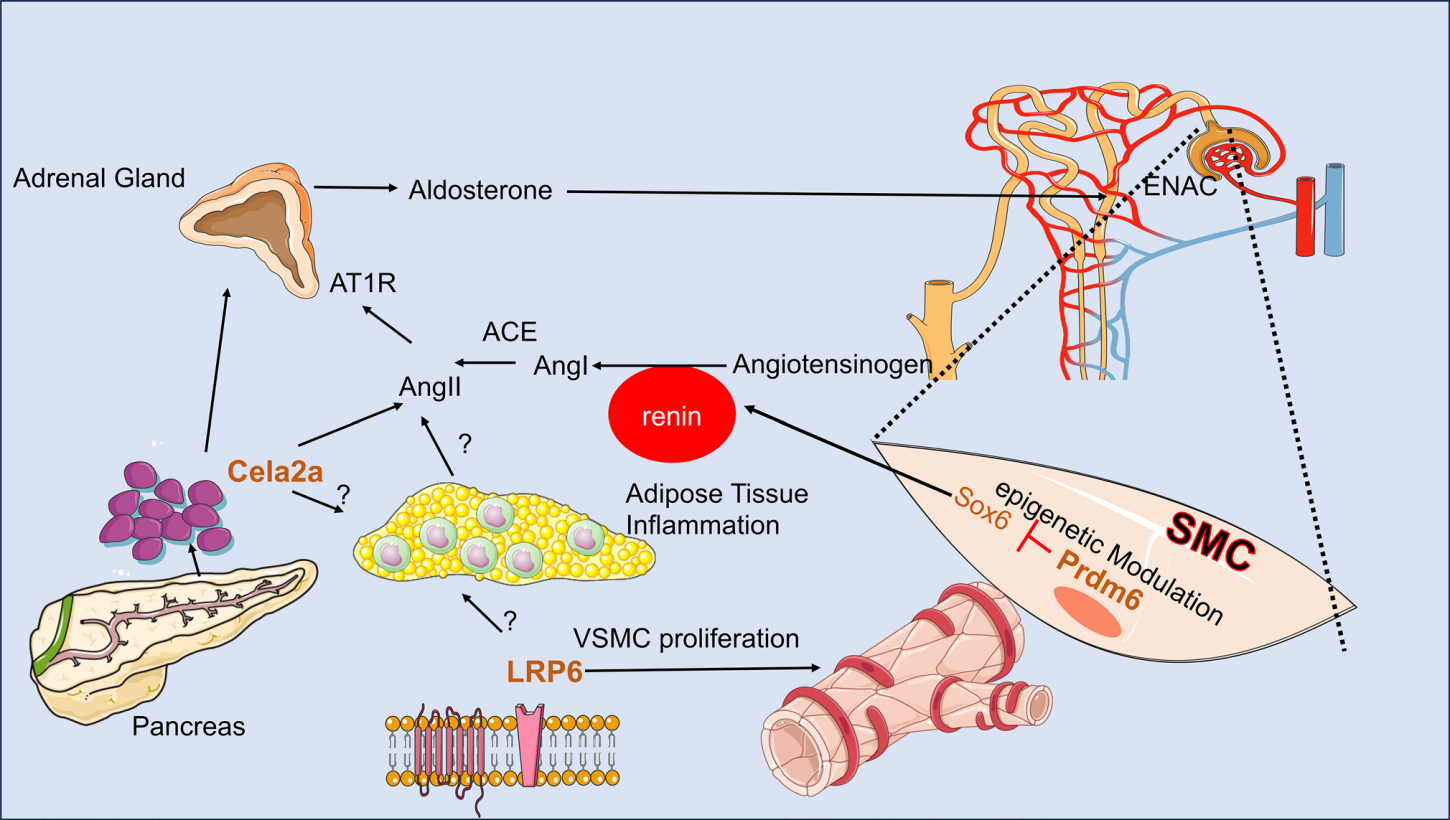
The potential mechanisms for hypertension by metabolic syndrome genes, *LRP6*, *PRDM6*, and *Cela2a* are shown. The latter 2 are genes at GWAS peaks for hypertension, while LRP6 is a gene underlying the monogenic form of hypertension

*Common variants of pancreatic exocrine chymotrypsin-like elastase 2A (CELA2A)* are associated with idiopathic hypertension. Using the unbiased approach of human genetics we have identified independent loss of function mutations in the gene encoding the pancreatic exocrine chymotrypsin-like elastase 2A (CELA2A), which contribute to early-onset atherosclerosis, impaired glucose tolerance/type2 diabetes (T2D), hypertension, hypertriglyceridemia, and obesity in outlier kindreds [45] (Fig. 1). This unprecedented discovery is anticipated to provide deep insight into novel pathways of blood pressure homeostasis, unravel novel disease risk factors and discover new targets for drug development. Notably, three common variants rs3820068 (p = 1 × 10–12), rs1042010 (p = 3 × 10–13), and rs3766160(2.09e-13) in the *CELA2A* locus exhibit genome-wide association with elevated blood pressure [46, 47], as reported by Cardiovascular Disease Knowledge Portal. These single nucleotide polymorphisms (SNPs) act as expression quantitative trait loci (eQTLs) for CELA2A in the adrenal gland, which exhibits high expression levels of this gene and encompasses all components of the renin–angiotensin–aldosterone system (RAAS) [46, 47]. It has been proposed that CELA2A regulates blood pressure by modulating angiotensin II levels (Fig. 2).

### Hypertension and metabolic syndrome

Hypertension is a key component of metabolic syndrome, a cluster of conditions that increase the risk of heart disease, stroke, and diabetes. Among the genetic factors linked to metabolic syndrome, rare variants in the CELA2A gene have been identified as associated with all its traits, including hypertension. Our research group was the first to discover the link between metabolic syndrome, severe hypertension, and the LRP6 gene [48]. We found rare nonconservative mutations in LRP6, which is crucial for encoding the Wnt coreceptor involved in regulating multiple bodily functions, in several families displaying autosomal dominant patterns of early coronary artery disease (CAD) and metabolic syndrome symptoms such as hyperlipidemia, hypertension, and diabetes [48, 49]. These discoveries underscored the impact of single-gene defects in Wnt signaling on CAD, hypertension, and other cardiovascular risk factors. Notably, the mutations were located in the second propeller domain of LRP6, essential for ligand binding, and were shown to segregate with metabolic syndrome traits in affected family members, but not in unaffected individuals. Functional analyses of these variants revealed a disruption in Wnt signaling pathways. Interestingly, common variants near R-spondin family member 3 (*RSPO3*) in chromosomal region 6q22 are associated with systolic (p = 2.56 × 10^−10^) and diastolic blood pressure 2.43 × 10^−11^). In addition, several SNPs at the hypertension locus on 3p22.1, including the index SNP rs1717027, have been associated with gene-expression levels of both *CTNNB1* in multiple tissues [50]. Future research utilizing transgenic mouse models aims to clarify whether hypertension that arises from aberrant Wnt signaling is primarily a vascular disorder or if it also involves alterations in renal salt and water balance. Considering the observed systemic vascular changes, it is plausible that vascular disease contributes, at least in part, to the development of hypertension.

In conclusion, exploring the genetic underpinnings of hypertension holds promise for identifying new targets for antihypertensive medication development.

## Data Availability

Not applicable.

## Abbreviations

11-β HSD2: 11β-Hydroxysteroid dehydrogenase-2
ACE: Angiotensin converting enzyme
ACTH: Adrenocorticotropic hormone
AME: Apparent mineralocorticoid excess
AngI: Angiotensin I
AngII: Angiotensin II
ASL: Argininosuccinate lyase
AT1R: Angiotensin II type I receptor
CAH: Congenital adrenal hyperplasia
CUL3: Cullin-3
CYP11B1: 11β-Hydroxylase gene
CYP11B2: Aldosterone synthase gene
FH: Familial hyperaldosteronism
GRA: Glucocorticoid-remediable aldosteronism
GWAS: Genome-wide association studies
HTN: Hypertension
HTNB: Autosomal hypertension with brachydactyly type e
KLHL3: Kelch-like 3
NEDD4: Neural precursor cell expressed developmentally down-regulated 4
NCC: Na^+^/Cl^−^ cotransporter
NKCC2: Na^+^–K^+^–Cl^−^ cotransporter 2
PDE3A: Phosphodiesterase 3A
RAAS: Renin–angiotensin–aldosterone system
SCNN1A, SCNN1B, SCNN1G: Epithelial sodium channel 1 subunit alpha, beta, and gamma
UMOD: Uromodulin
VSMC: Vascular smooth muscle cells
Zona F.: Zona Fasciculata
Zona G.: Zona glomerulosa
